# Hydrophobia

**Published:** 1889-06-08

**Authors:** 


### HYDROPHOBIA.

*(Continued from page 130.)*

It is well known that all persons are not equally susceptible to all poisons, or to other diseases, and it is not consistent with such knowledge to suppose hydrophobia an exception, and that the rabies virus is equally powerful at all times in every temperament and constitution. It is a fact that all dogs are not susceptible to rabies. Again, it is quite possible that the quality of the virus may vary in different animals, at different times of life, and under different circumstances. Also, a person may be injured by a rabid animal, yet no virus may enter the wound, for it may be deposited on clothing through which the fang or claw* inflicting the wound passes. It is also quite possible that the virus may be expelled from the wound if copious bleeding takes place. An instance was mentioned by Hunter of twenty-one persons being bitten by the same mad dog, of whom only one suffered. Troillett gives another example, when seventeen were bitten and only one suffered. Watson quotes a further instance of three only suffering out of fifteen bitten. Of 114 bitten by rabid wolves, sixty-seven were victims. Dr. Bristowe (*British Medical Journal*, April 21, 1883) remarks that only a minority of the persons bitten by mad dogs contract hydrophobia; and Dr. Gowers, in "Quain's Dictionary," and also in his recent work on "Diseases of the Nervous System," states, when no preventive measures are adopted, at least half —perhaps two-thirds—of persons bitten escape. Dr. Gowers also says, in "Diseases of the Nervous System," it has been calculated (by Bolinger) that of those bitten by certainly rabid dogs 47 per cent. suffer. Of those whose wounds were uncauterised 83 per cent., but of those promptly cauterised only 33 per cent. Zeimessen makes a similar statement. And it is mentioned by Erichsen that Dr. White, of Brighton, disbelieving in the contagious nature of the disease, inoculated himself with a rabid dog's saliva with impunity. Chevers, in his "Commentary on Indian Diseases," mentioned a case of fourteen persons bitten and treated by washing and cauterisation of the wound, all remaining well at the end of fifteen months, but three bitten at the same time, and not treated, died. It may, therefore, be safely asserted that it does not necessarily follow that a person bitten by a mad dog must get hydrophobia. This frequent immunity from the disease of persons bitten has tended to confer reputation on many vaunted methods of prevention. So long as it cannot be stated that a person bitten by a dog will certainly have hydrophobia, his not having hydrophobia cannot certainly be attributed to any preventive means used.

There are yet other popular fallacies, one of which is that all persons contracting hydrophobia must die—that the disease is necessarily fatal. While the extremely dangerous character of the disease may be freely admitted, an inevitable fatal sequence is neither in accord with our knowledge of other maladies, nor with creditable evidence. There is probably no poison, not even cobra venom, which may not be introduced into the system in quantities only sufficient to cause illness, not death. The old adage, "While there is life there is hope," is certainly correct so far as regards acute maladies, and there is no acute disease from which an occasional recovery does not take place. Hydrophobia is not an exception, as is generally supposed. This has been noted from early times. Avicenna says, "Cura propinqua est ante terrorem aquæ," and Baungmarten says, "Before the dread of water sets in, the cure is not only practicable, but not unfrequent." Cases of recovery have been reported by Offenburg under curara, from America by the same drug, by Dr. Nichols under morphia and Calabar bean; in the *Medical and Physical Transactions, Bombay*, for 1860-61; in *Indian Annals of Medicine*; in the *Lancet* for 1872, and in several other places. Those who believe hydrophobia must certainly be fatal are apt to regard cases of recovery as pseudo-hydrophobia. We have, however, witnessed two casess of recovery. There is also an erroneous impression that hydrophobia is a very prevalent malady. This arises in a great degree from nearly every instance being chronicled in the newspapers. As a matter of fact, hydrophobia is comparatively unfrequent. It has already been stated that all persons injured by mad dogs do not suffer from hydrophobia. During the ten years ending 1885, the average annual number of deaths from hydrophobia in England was forty-three, which probably represents a total number of 800 bitten by rabid dogs. It thus happens that many medical men never even meet with a case of hydrophobia.

Certain other popular fallacies are connected with hydrophobia, one being the pre-eminently horrible nature of the malady. It may, however, be questioned if death from hydrophobia, however dreadful it may be, is more terrible than death from several other causes. Tetanus, for example, in which the spasms are tonic or continuous, without the intervals of relaxation occurring in hydrophobia. Some forms of internal cancer entail a lingering, painful end, to be dreaded as much as hydrophobia. In former days, persons with hydrophobia were shunned and abandoned by even their nearest relatives. Within the century, people with hydrophobia are said to have been shot in Austria. Here, they used to be smothered in feather beds. A woman told Henry Kingsley that her mother had assisted at one of these immolations. Doubtless the hazy knowledge of such procedures has tended to impress the public mind with the horrible nature of the malady, transcending all other causes of death. Then there is the impression that the patient with hydrophobia barks and bites like a dog. If this ever occurs it is very seldom, and probably the "hawking" up of the saliva and the spasmodic movements of the jaws have given rise to the idea. It is true that the mental distress always present during hydrophobia sometimes amounts to frenzy. The sufferer has been known to rush wildly about the apartment in a state of maniacal frenzy and clawing at the throat. Sometimes he appeals for restraint lest he may obey the insane impulse to injure someone. But this he does not attempt to do by biting. Beaumetz states that whenever such symptoms are produced, the medical attendant has to deal with a case of alcoholism, and not with hydrophobia.

It would be an useless task to mention the numerous remedies, both absurd and physiologically reasonable, which have been proposed as more or less "certain cures" for hydrophobia. Such proposals range from the blood of a spoon bill duck to the powdered skull of a still-born infant, from hot vapour baths to cold and ice, and comprise every powerful drug both in and out of the pharmacopœia. And when perchance a case of recovery apparently occurred under any treatment, the means employed has generally proved worthless on the next occasion. This, however, is not peculiar to hydrophobia, for the same may be said of cholera and of several other affections. As with snakebite in India, so with hydrophobia in Europe, the disease has always presented a field for the amateur doctor or the professional charlatan. One of the most recent examples of professing the possession of a cure for hydrophobia is that of a person named McGavern, in the west of Cavan, Ireland, who was taken up by a board of guardians, and on whose treatment a committee was proposed by the Vice-President

* Although the saliva is the secretion containing the poison, a wound made by a claw may cause hydrophobia, should saliva be on the claw.

of the Irish College of Surgeons (*British Medical Journal*, December 3, 1887). It was afterwards proved that McGavern's treatment was not successful, and doubtless his reputation depended on the facts that all bitten by a mad dog do not suffer, and that others bitten suffer from the pseudo-form of the malady from which recovery is the rule. An old Tamil proverb has it that "the bite of a dog needs the use of the sandal." The first thing done in some parts of the East is to beat the part injured with a sandal or shoe. This is an article always at hand, and of a suitable shape, and probably the reputation of the practice depends on its causing the wound to bleed freely by which the virus may be washed out.

The idea of the horrible nature of hydrophobia, and of its certain fatal termination, being so strongly fixed on the public mind, when M. Pasteur with his great reputation announced a preventive cure, it was received with acclamation and without question by the public. Persons bitten by dogs rushed off to Paris—not only from European countries, but also from America and from the East—anxious to place themselves under M. Pasteur's treatment, although the great majority were not aware of what the treatment consisted of. Still, great ignorance prevails among the public as to Pasteur's system. It is dependant on the fact that by drying the spinal cord of rabbits which have been rendered rabid by inoculation, the virus becomes less active, and the subcutaneous injection of an emulsion of such cord dried for fourteen days causes no definite symptoms of the disease, nor do subsequent inoculations or injections made with cords dried for a less time. After such inoculation an animal is protected, and does not suffer from rabies if afterwards bitten by a rabid animal. Pasteur further believes that such a course of injections is effective in preventing the occurrence of the disease in a person who has already been inoculated with rabies. It will thus be observed that Pasteur's system is not precisely the same as vaccination. In the one case, vaccination, there is usually no previous introduction of poison into the body. The inoculation for prevention of hydrophobia is performed after the introduction of a poison into the system.

*(To be continued.)*

